# Aldehyde Dehydrogenases and Prostate Cancer: Shedding Light on Isoform Distribution to Reveal Druggable Target

**DOI:** 10.3390/biomedicines8120569

**Published:** 2020-12-04

**Authors:** Luca Quattrini, Maria Sadiq, Giovanni Petrarolo, Norman J. Maitland, Fiona M. Frame, Klaus Pors, Concettina La Motta

**Affiliations:** 1 Department of Pharmacy, University of Pisa, Via Bonanno 6, 56126 Pisa, Italy; luca.quattrini@farm.unipi.it (L.Q.); giovanni.petrarolo@phd.unipi.it (G.P.); 2 Institute of Cancer Therapeutics, School of Pharmacy and Medical Sciences, Faculty of Life Sciences, University of Bradford, West Yorkshire BD7 1DP, UK; M.Sadiq@bradford.ac.uk; 3 Cancer Research Unit, Department of Biology, University of York, Heslington, North Yorkshire YO10 5DD, UK; n.j.maitland@york.ac.uk (N.J.M.); fiona.frame@york.ac.uk (F.M.F.); 4 CISUP, Centre for Instrumentation Sharing, University of Pisa, Lungarno Pacinotti 43, 56128 Pisa, Italy

**Keywords:** prostate cancer, aldehyde dehydrogenase, ALDH1A1, ALDH1A3, ALDH inhibitors, imidazo[1,2-*a*]pyridines

## Abstract

Prostate cancer represents the most common malignancy diagnosed in men, and is the second-leading cause of cancer death in this population. In spite of dedicated efforts, the current therapies are rarely curative, requiring the development of novel approaches based on innovative molecular targets. In this work, we validated aldehyde dehydrogenase 1A1 and 1A3 isoform expressions in different prostatic tissue-derived cell lines (normal, benign and malignant) and patient-derived primary prostate tumor epithelial cells, demonstrating their potential for therapeutic intervention using a small library of aldehyde dehydrogenase inhibitors. Compound **3b**, 6-(4-fluorophenyl)-2-phenylimidazo [1,2-*a*]pyridine exhibited not only antiproliferative activity in the nanomolar range against the P4E6 cell line, derived from localized prostate cancer, and PC3 cell lines, derived from prostate cancer bone metastasis, but also inhibitory efficacy against PC3 colony-forming efficiency. Considering its concomitant reduced activity against normal prostate cells, **3b** has the potential as a lead compound to treat prostate cancer by means of a still untapped molecular target.

## 1. Introduction

Prostate cancer (PCa) represents the most common non-cutaneous malignancy diagnosed in men, with more than 1,200,000 new estimated cases each year and over 350,000 deaths worldwide [1]. Although it can be successfully treated in its early stage with radiation therapy and radical prostatectomy, once it has escaped the prostate gland treatment is mainly by using androgen deprivation therapy (ADT). PCa now represents the second-leading cause of cancer death for men [2,3]. Patients who no longer respond to ADT develop an aggressive and often untreatable form of PCa known as castrate-resistant prostate cancer (CRPC), which is characterized by a high propensity for metastasis and short median survival rates ranging from 12.1 to 27.0 months [4,5,6]. Drugs that are used to treat advanced stages of PCa include the androgen receptor (AR) inhibitor enzalutamide [7], the CYP171A1 inhibitor abiraterone acetate [8], the taxanes docetaxel [9] and cabazitaxel [10], the radioactive isotope Radium-223 dichloride [11] and sipuleucel-T, which is an autologous cellular immunotherapy manufactured from antigen-presenting cells [12]. Unfortunately, these therapies are rarely curative necessitating the need for the identification of new molecular targets and/or development of therapeutic strategies to treat aggressive PCa. Although advances in the former have been made using genomic and transcriptomic sequencing as well as clonal tracking [13], the PCa microenvironment is complex and plays host to a number of different cell types including subpopulations of cells endowed with tumor-initiating capability and compartments under hypoxic stress, which both impact on response to drug treatments [14,15]. Prostate cancer stem cells (PCSCs) that possess tumor-initiating capacity represent a small percentage of the whole cancer population, yet these are considered to play a major role in patient relapse. Indeed, CSCs are known to increase DNA repair capacity, drug efflux system, and resistance to reactive oxygen species (ROS), which make them refractory to the common cancer treatments, resulting in more aggressive phenotypes. Accordingly, treatment regimens with improved efficacy are believed to benefit from the inclusion of a therapeutic aimed at eradicating PCSCs [16,17,18].

Various cell surface proteins, including CD44 [19], α2β1 integrin [20], and CD133 [21], as well as enzymes like aldehyde dehydrogenases (ALDHs) [22], have been useful in helping to identify CSC populations. Besides playing a key role as a molecular marker for tracking stem-like cells within the tumor bulk, ALDHs have also been linked to chemo- and radio-resistance [23,24] while their expression provides an opportunity for therapeutic intervention [25]. Members of the ALDH1A family seem to be important in many cancer types, including PCa, where both ALDH1A1 and 1A3 isoforms have been reported to be expressed at higher levels in tumor tissue compared to benign prostatic hyperplasia and normal prostate [26] while 1A2 may have value as a tumor suppressor gene [27]. Moreover, they are also acknowledged to promote clonogenic and migration cell capabilities in vitro and enhance the metastatic potential in vivo [28] while expression also correlates with higher Gleason score (G8–9) in vivo [29]. In addition to the ALDH1A members, other isoforms have also been shown to be expressed in PCa samples, including ALDH4A1, 7A1, 9A1, and 18A1 [26,28], which indicates a complex and challenging picture of unravelling functional roles of each individual isoform.

In this study we sought to validate ALDH1A1 and 1A3 isoform expression as an opportunity to demonstrate their potential for therapeutic intervention using a small library of novel selective ALDH inhibitors [30,31]. Functional efficacy of the compounds was assessed in both established prostate cell lines, as well as patient-derived primary prostate tumor epithelial cells.

## 2. Experimental Section

### 2.1. Chemistry

#### 2.1.1. Materials and Methods

MW assisted reactions were carried out in a Biotage^®^ Initiator+ Microwave Synthesizer (Biotage, Uppsala, Sweden). Melting points were determined using a Reichert Köfler hot-stage apparatus (Reichert Technologies, Depew, NY, USA) and are uncorrected. Routine ^1^H-NMR and ^13^C-NMR spectra were recorded in DMSO-d6 solution on a Bruker 400 spectrometer operating at 400 MHz. Evaporation was performed in vacuo (rotary evaporator). Analytical TLCs were carried out on Merck 0.2 mm precoated silica gel aluminium sheets (60 F-254) (Merck Millipore, Burlington, MA, USA). Purity of the target inhibitors was determined by HPLC analysis, using a Shimadzu LC-20AD liquid chromatograph (PDA, 250–500 nm, Shimadzu, Kyoto, Japan) and a Luna^®^ C18 column (250 mm × 4.6 mm, 5 μm (Phenomenex, Torrance, CA, USA), with a gradient of 30% water and 70% acetonitrile and a flow rate of 1.0 mL/min. All the compounds showed percent purity values ≥95%. HRMS were obtained with a Q Exactive™ Plus Hybrid Quadrupole-Orbitrap™ Mass Spectrometer (Thermo Fisher Scientific, Waltham, MA, USA). 5-Bromopyridin-2-amine, 2-bromo-1-phenylethan-1-one, and the appropriate boronic acids, used to obtain the target inhibitors **3a–d** as depicted in Figure S1, were from Activate Scientific (R&D Chemicals, Regensburg, Germany). 

#### 2.1.2. Synthesis of 6-Bromo-2-phenylimidazo [1,2-*a*]pyridine, **2**

A mixture of 5-bromopyridin-2-amine 1 (1.00 mmol), 2-bromo-1-phenylethan-1-one (1.00 mmol) and sodium bicarbonate (1.00 mmol) in water was allowed to react under stirring and microwave heating in a sealed vial, at 100 °C for 30 min. After cooling, the obtained was collected by filtration, then purified by recrystallization from EtOH and characterized with physio-chemical and spectroscopic data [30]. Figure S1: Synthetic procedure used to derive (substituted)imidazo[1,2-*a*]pyridine derivatives **3a–d**.

#### 2.1.3. General Procedure for the Synthesis of 2,6-(Substituted)diphenylimidazo[1,2-*a*]pyridines, **3a–d**

A solution of 6-bromo-2-phenylimidazo[1,2-*a*]pyridine 2 (1.00 mmol), Pd(OAc)_2_ (0.10 mmol), and PPh_3_ (0.20 mmol) in ethanol was left under stirring at room temperature for 30 min, then added with the suitable phenyl boronic acid (1.50 mmol), dissolved in ethanol, and 2 mL of Na_2_CO_3_ 2 M. The resulting mixture was refluxed under stirring until the disappearance of the starting material (TLC analysis). After cooling, the crude obtained was evaporated to dryness under reduced pressure, then purified by column chromatography (silica gel, ethyl acetate/petroleum ether). The pure product **3a–d** was recrystallized from the suitable solvent and characterized by physio-chemical and spectroscopic data [30].

### 2.2. Biology

#### 2.2.1. Materials and Methods

Five different prostate cell lines, including normal cell line PNT2-C2 (Merck), benign prostatic hyperplasia cell line BPH1 (gift from Simon W. Hayward, Evanston, IL, USA), and cancer cell lines including PC-3 (derived from prostate cancer bone metastasis) (ATCC), LNCaP (derived from prostate cancer lymph node metastasis) (ATCC), and P4E6 (derived from localized prostate cancer) (derived in York and available from European Collection of Authenticated Cell Cultures ECACC), as well as two primary malignant cell lines, H796/19 and H798/19 (both Gleason 7 grade cancers) (obtained in York with ethical permission, REC ref 07/H1304/121) were used in the study. Culturing of commercially available cell lines [32] and processing and culturing of primary cells [33] were carried out as previously described. Primary cells were typically used at a passage <5 since they have finite growth and the intention is to maintain them as close to the original tumor as possible.

#### 2.2.2. Protein Extraction

Cells were harvested using trypsin and the resulting pellets were lysed in CytoBuster lysis buffer (Merck Millipore, Burlington, MA, USA) with the addition of protease inhibitors (cOmpleteTM, Mini, EDTA-free Protease Inhibitor Cocktail (Roche, Basel, Switzerland) and Phosphatase (PhosSTOP) (Roche, Basel, Switzerland). Cells lysed in CytoBuster were incubated on ice for 5 min and then centrifuged at 13,000 RPM for 5 min. The supernatant was then transferred into a new 1.5 mL microcentrifuge tube as the whole cell lysate.

#### 2.2.3. Protein Quantification

A bicinchoninic acid (BCA) assay (Thermo Fisher Scientific, Waltham, MA, USA) was used to quantify protein concentration from whole-cell lysates according to the manufacturer’s instructions. Standards of known concentrations of BSA were made in the same lysis buffer as the unknown samples. Amounts of 10 μL of each standard or unknown sample were added to a 96 well plate in triplicate. An amount of 200 μL of the pre-made BCA assay working solution was then added to each well and the plate was incubated at 37 °C for 30 min. The plate was cooled down to room temperature and then read on a POLARstar OPTIMA microplate reader (BMG Labtech, Aylesbury, Bucks, UK) for absorbance at 562 nm. A standard curve was generated from the BSA standards and protein concentration of unknown samples was calculated from the line of best fit.

#### 2.2.4. SDS-PAGE Gel Electrophoresis

10% Tris-SDS acrylamide gels were prepared using the Bio-Rad protean II system (Bio-Rad Laboratories, Hercules, CA, USA). An amount of 30 μg of protein lysate was added to 4× Laemmli sample buffer (Bio-Rad) and heated to 95 °C for 5 min. Up to 30 μL of samples were added to the wells with the Precision Plus Protein kaleidoscope ladder (Bio-Rad Laboratories, Hercules, CA, USA) in a separate lane to determine the size of proteins. Proteins were subjected to electrophoresis at 80 V for 2 h.

#### 2.2.5. Western Blot

Immobilon-P membrane (Merck Millipore, Burlington, MA, USA) was activated by immersion in methanol for 30 s and washed in dH_2_O. Gels were placed onto the membrane and transferred using the Bio-Rad Protean II system in transfer buffer (48 mM Tris, 39 mM glycine, 10% (*v*/*v*) methanol) at 40 V overnight. Membranes were then blocked with 5% (*w*/*v*) non-fat skimmed milk (Marvel) at room temperature for 1 h. Primary antibody (Table 1) diluted in 1% (*w*/*v*) Marvel in TBST (150 mM NaCl, 50 mM Tris-HCl pH 7.5, 0.1% (*v*/*v*) Tween 20) was added and incubated overnight at 4 °C. The following day, membranes were washed in TBST buffer three times for 5 min. Membranes were incubated with secondary antibody (Table 1) for 1 h at room temperature. After washing in TBST three times for 5 min, the BM Chemiluminescence Blotting Substrate (Roche, Roche, Basel, Switzerland) was used to develop the membranes. Solution A was added to Solution B at a dilution of 1:100 and added to the membrane for 1 min. The excess was removed, and the membranes were exposed to hyperfilm ECL (GE Healthcare, Chicago, IL, USA) and processed using an X-ray processor (SRX-101A, Konica Minolta). 

#### 2.2.6. Immunofluorescence

Cells were plated onto 8 well chamber slides and left to adhere overnight (~10,000 cells/well). Falcon culture slides were used (Corning, NY, USA). Following two PBS washes, cells were then fixed with 200 μL 4% paraformaldehyde (PFA) pH 7.4 for 15 min at room temperature and washed again with PBS. Cells were then blocked in 5% (*v*/*v*) goat serum in PBS with 0.3% of Triton X-100 for 1 h at room temperature. Cells were then incubated with primary antibodies (Table 2) diluted 1:400 in 1% goat serum in PBS with 0.3% of Triton X-100 overnight at 4 °C. Secondary antibody only controls were performed by incubating in 1% goat serum only overnight. The following day, slides were washed three times in PBS for 5 min and incubated with 200 μL secondary antibody (Table 2) in 1% goat serum for 1h in the dark. Cells were washed a final three times with PBS for 5 min whilst protected from light and the chambers were then removed. Nuclear staining was performed using Vectashield mounting medium with 4′,6-diamidino-2-phenylindole (DAPI) (Vector Laboratories, Burlingame, CA, USA) and slides covered with a coverslip (22 × 50 mm) (Scientific Laboratory Supplies Ltd., Nottingham, UK) and sealed with clear nail varnish. Slides were analyzed on a Leica DMIL LED fluorescent microscope. 

#### 2.2.7. Cell Viability

AlamarBlue quantitatively measures cell viability since actively metabolizing cells can reduce its active ingredient resazurin to a fluorescent molecule (resorufin) which can be subsequently analyzed on a plate reader. Cells were plated in 96 well plates at a density of 5000 cells/well and left to adhere overnight in 200 μL media. The following day cells were treated with 9 different concentrations of the test drugs in triplicate, ranging from 10^−5^ M to 10^−13^ M. After 72 h exposure 20 μL of AlamarBlue reagent (diluted 1:10 in the corresponding media for each cell line) was added to each well and incubated at 37 °C for 2 h. Fluorescence intensity was determined using a microplate reader (Polarstar Optima, BMG Labtech) at excitation/emission values 73 of 544/590 nm. EC_50_ values were calculated using the software GraphPad Prism Version 6 (San Diego, CA, USA).

#### 2.2.8. Colony-Forming Assay

PNT2-C2, BPH1 and PC3 cell lines were plated in 12-well plates with a density of 4 × 10^4^ cells/well and left to adhere overnight in 1.00 mL media. The following day cells were treated with 3 different concentrations of the test drugs, based on the EC_50_ values in cell viability (EC_50_, EC_50×_2, EC_50×_5). After 72 h of exposure, cells were counted and plated into 12 well plates in triplicate with a density of 100 cells/well. At day 8, cells were stained with crystal violet (1% (*w*/*v*) crystal violet, 10% (*v*/*v*) ethanol in PBS). Colonies consisting of >32 cells were counted (representative of 5 population doublings).

#### 2.2.9. Real-Time Quantitative PCR

RNA extraction was carried out using the RNeasy Mini Kit (Qiagen, Hilden, Germany). The concentration and quality of the eluted RNA were determined using a NanodropTM2000 (Thermo Fisher Scientific, Waltham, MA, USA) spectrophotometer and measuring the 260/280 ratio. Total RNA of 50–2000 ng was reverse transcribed into single-stranded cDNA using the High Capacity cDNA Reverse Transcription Kit (Thermo Fisher Scientific, Waltham, MA, USA). Once the reaction finished, samples were purified using the QIAquick PCR Purification Kit (Qiagen). The concentration and quality of the cDNA was measured by using the NanodropTM2000 spectrophotometer. qPCR was carried out in 25 μL total PCR reaction using the TaqMan Universal PCR Master Mix (Applied Biosystems, Thermo Fisher Scientific, Waltham, MA, USA). The PCR reaction consisted of 12.5 μL of 2× master mix, 1.25 μL of 20× TaqMan Gene Expression Assay Mix (Supplementary Table S1), and 11.25 μL cDNA diluted in dH_2_O. A 96-well MicroAmp Optical plate (Applied Biosystems) was used and all reactions were run in triplicates. Primers used were obtained from TaqMan Gene Expression Assays (Thermo Fisher Scientific, Waltham, MA, USA). The PCR reactions were centrifuged and then run on 7500 Real time PCR system and analysis was carried out using the 7500 software v2.3 (Applied Biosystems). The thermal cycling conditions consisted of an initial setup of a hot start of 10 min at 95 °C which was followed by 40 cycles of 15 s at 95 °C for denaturing and 1 min at 60 °C for annealing/extending. The gene expression level relative to internal control RPLP0 was calculated using the formula 2^−∆CT^ and the fold change in gene expression was worked out using the 2^−∆∆CT^ method.

#### 2.2.10. Quantification and Statistical Analysis

All statistical analyses were produced using Prism 8 (GraphPad Software, San Diego, CA, USA). Details regarding statistical tests are reported in Figure Legends and Supplemental Information.

## 3. Results and Discussion

### 3.1. ALDH Expression Analysis

**Figure 1:**
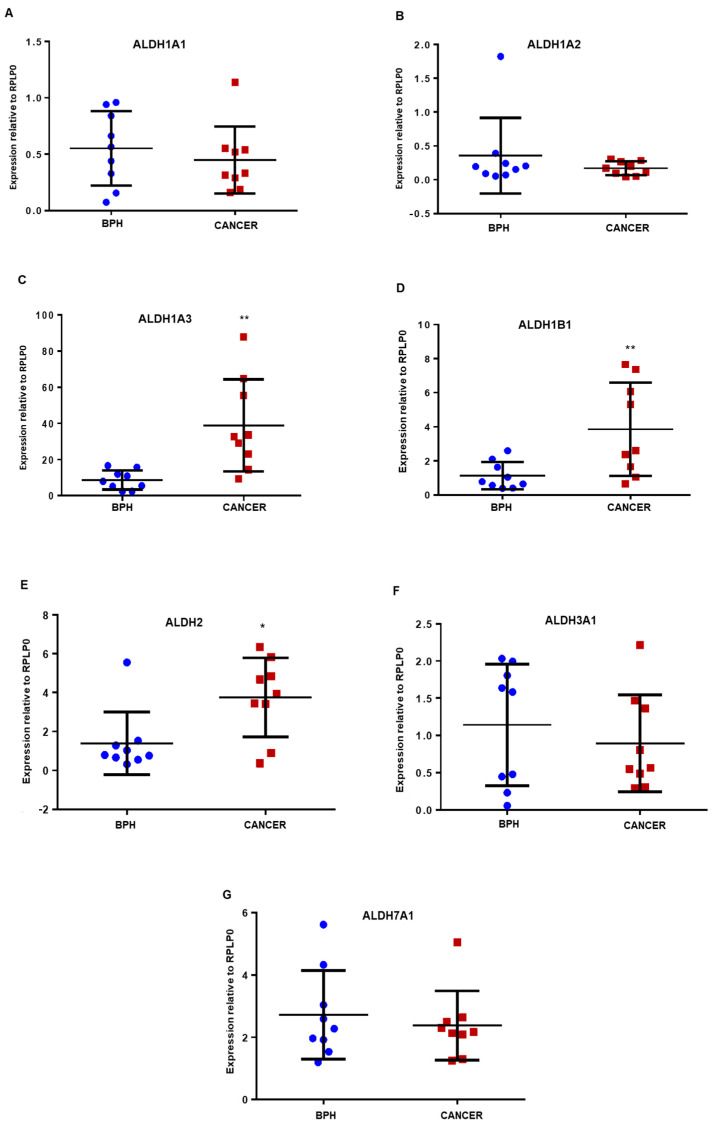
qPCR analysis of ALDH gene expression relative to RPLP0 in prostate primary epithelial cultures. Gene expression of (**A**) ALDH1A1, (**B**) ALDH1A2, (**C**) ALDH1A3, (**D**) ALDH1B1, (**E**) ALDH2, (**F**) ALDH3A1 and (**G**) ALDH7A1 was measured using 2^−∆CT^. RNA was extracted from patient-derived prostate epithelial cells from prostate cancer tissue (*n* = 9) and benign prostatic hyperplasia (BPH) tissue (*n* = 9). * Note difference in scale on *Y*-axis. Statistical significance was calculated using Mann–Whitney U test, for unpaired groups, non-parametric distribution, comparison of only two groups. BPH samples denoted as blue circles and cancer samples as red squares. * *p* = 0.01 to 0.05, ** *p* = 0.001 to 0.01.

**Figure 2:**
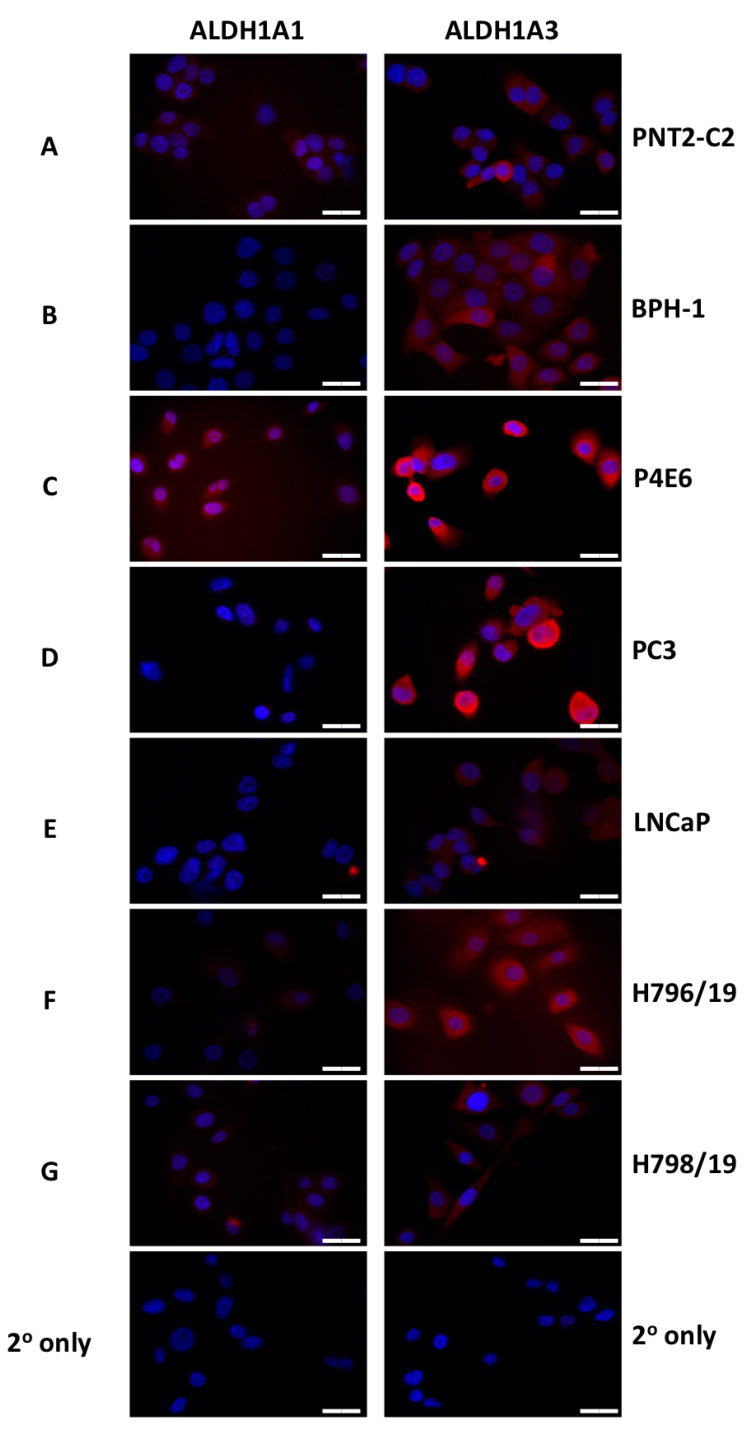
Immunocytochemical staining of ALDH1A1 and ALDH1A3 within different prostate cell lines and patient-derived primary epithelial cells. (**A**) PNT2-C2, normal prostate epithelial cell line; (**B**) BPH-1, benign prostatic hyperplasia cell line; (**C**) P4E6, differentiated prostate cancer cell line; (**D**) PC-3, prostate cancer cell line derived from bone metastasis; (**E**) LNCaP, prostate cancer cell line derived from lymph node metastasis; (**F**) H796/19, patient-derived malignant epithelial cell line; (**G**) H798/19, patient-derived malignant epithelial cell line; BPH1, benign prostatic hyperplasia cell line. (Scale bar = 20 µm).

Analysis of mRNA expression in nine benign prostatic hyperplasia (BPH) and nine malignant PCa samples derived from patients with a Gleason score ranging from 6–9 revealed significant ALDH isoform differences (Figure 1). Specifically, the expression of ALDH1A3 (Figure 1C), 1B1 (Figure 1D) and 2 (Figure 1E) was observed to be higher in primary prostate cancer samples than in BPH samples, with ALDH1A3 levels being notably much higher compared to that of other isoforms. ALDH1A1, 3A1 and 7A1 were found to be similarly expressed in benign and cancer samples (Figure 1A,F,G, respectively). The expression of ALDH1A2 was low in most samples analysed (Figure 1B) and is in accordance with previous findings that indicated it to be epigenetically silenced in malignant tissue [27].

High ALDH activity has been linked to PCa subpopulations with a propensity for being tumorigenic, but there is no information on which isoform is present in PCSCs. Using a small cohort of samples we performed qPCR analysis of primary prostate epithelial cells, which had been selected into a stem cell (SC), transit-amplifying (TA), and committed basal (CB) cell populations based on their cell surface antigens [34]. Due to small sample size and in some cases low or variable RNA extraction yield, no statistical difference in ALDH expression between any sub-populations was observed. Other studies that showed ALDH expression in stem cells measured protein, whereas this experiment was measuring RNA; it is possible that they would not directly correlate. However, relative ALDH isoform expression in accordance with the whole population primary cell data (Figure 1) was seen. ALDH1A2 was the least expressed in most samples (apart from one outlier) compared to other isoforms while ALDH1A3 expression was consistently highest followed by ALDH1A1 and 7A1 in some samples (Figure S2).

The ALDH1A isoform members have generated considerable interest, and our own analysis indicates differential expression of these isoforms. While ALDH1A2 seems to act as a tumor suppressor gene with low expression in PCa, ALDH1A1 and 1A3 have frequently been shown to be expressed in CSC populations and in PCa may contribute to malignancy [26]. Accordingly, we next evaluated 1A1 and 1A3 isoforms in a panel of prostate cancer cell lines (P4E6, PC-3, LNCaP), patient-derived primary prostate epithelial cells (H796/19 and H798/19) obtained from radical prostatectomies, a normal prostate epithelial cell line (PNT2-C2) and a benign prostatic hyperplasia cell line (BPH). The purpose of the expression profiling was to identify a panel of cell lines with ALDH1A1 and/or 1A3 target expression to investigate the potential of a select group of ALDH1A-targeting compounds. Using Western blot analysis, the 1A1 isoform was more highly expressed in P4E6, PC-3 and primary H796/19 and H798/19 lines, compared to the normal PNT2-C2 and benign BPH1; no expression was observed in LNCaP (Figure S3). In contrast, ALDH1A3 was expressed in all the samples, with elevated protein expression levels in the P4E6 and PC-3 cell lines (Figure S3). Immunocytochemistry analyses were also performed, to clarify ALDH1A1 and 1A3 expression and distribution within the various cell types (Figure 2). ALDH1A3 was again shown to be more highly expressed in P4E6 and PC-3 cell lines (Figure 2C,D), correlating with the protein expression indicated by Western blotting while ALDH1A1 was also highly expressed in the P4E6 cell line. Image analysis (Figure S4) indicated that ALDH1A3 was primarily expressed in the cytoplasm, with some expression in the nucleus and ALDH1A1 was overall less expressed but with indications of some nuclear expression. In order to do a full analysis of cellular localisation of both isoforms, confocal microscopy should be used along with nuclear and cytoplasmic extraction protocols for Western blotting.

### 3.2. ALDH Inhibitors of the Imidazo[1,2-a]pyridine Series Have Anti-Proliferative Effects against Different Prostatic Tissue-Derived Cell Lines

Representative examples of ALDH inhibitors from our in-house collection of compounds, selected among those showing the best inhibitory properties against the targets ALDH1A1 and 1A3 [30], were investigated for their anti-proliferative activity in malignant, benign and normal epithelial cell lines. After 72 h of exposure, all the compounds exhibited antiproliferative activity in the nanomolar (nM) range (EC_50_: ~5–425 nM) in a dose-dependent manner, as measured using the Alamar Blue assay (Table 3). The P4E6 cell line expressing high levels of both ALDH1A1 and 3A1 was the most sensitive to treatment with the panel of compounds, indicating potential target engagement that correlates with antiproliferative activity. Derivative **3b**, bearing a 4-fluoro atom on the pendant 6-phenyl ring, was the most potent analogue (EC_50_ 4.038 nM and 70.92 nM against P4E6 and PC3, respectively) with reduced activity in LNCaP (EC_50_ 240 nM) and the normal epithelial PNT2-C2 (EC_50_ 217 nM) cell line.

**Figure 3:**
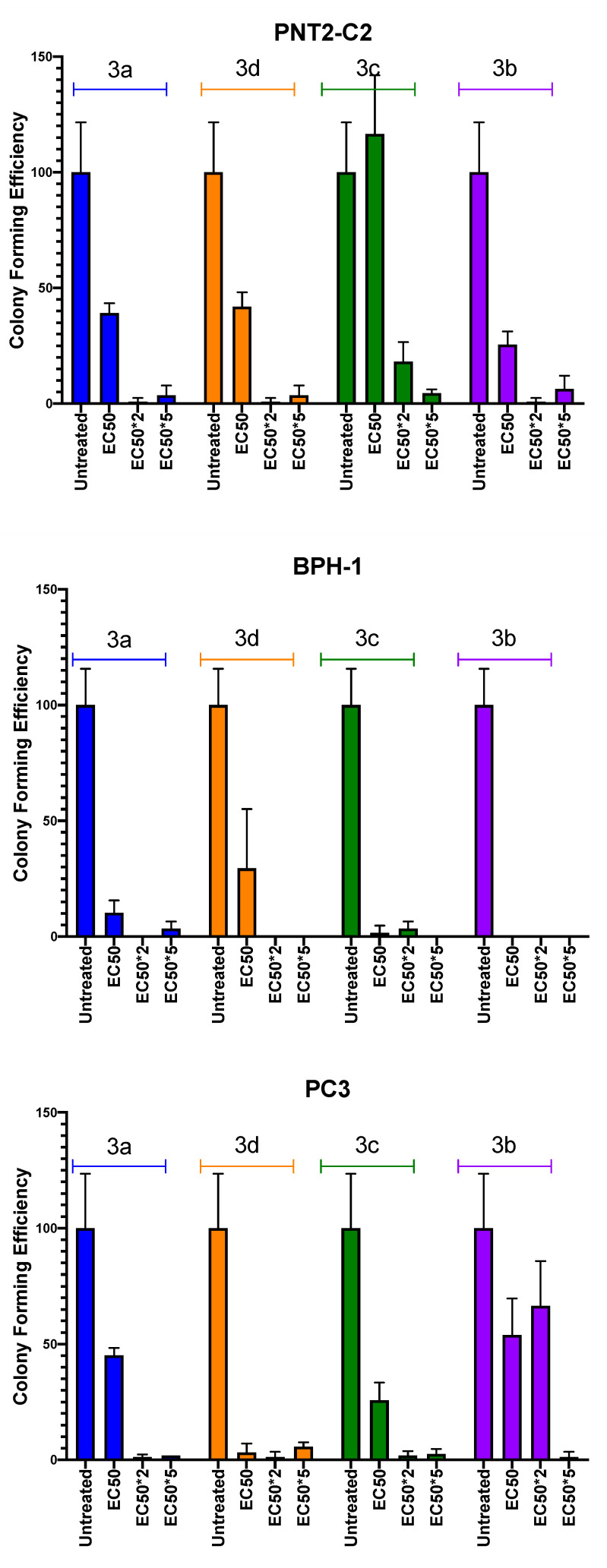
Colony-forming efficiency of selected cell lines in the presence of imidazo[1,2-*a*]pyridine derivatives. Colony-forming efficiency of PNT2-C2, BPH-1 and PC3 cell lines treated with **3a** (blue), **3b** (purple), **3c** (green) and **3d** (orange) at EC_50_, EC_50×_2, and EC_50×_5 test concentration.

In a recent study, the colony-forming efficiency was demonstrated to be strictly correlated to ALDH activity in a PCa cell population [28]. Accordingly, derivatives **3a–d** were also investigated for their ability to inhibit this cell property. PNT2-C2, BPH-1 and PC3 cell lines were exposed for 72 h to three different compound concentrations, EC_50_, EC_50×_2, and EC_50×_5, then plated in vitro at low density to allow colony formation. As shown in Figure 3, colony-forming efficiency was significantly reduced in all the treated samples, and almost nullified at the highest EC_50×_2 and EC_50×_5 investigated doses. Whilst **3b** was the most potent in the Alamar blue assays, compound **3d** was the only one that selectively inhibited PC3 colony forming more strongly than PNT2-C2 or BPH-1. The ideal drug candidate is one that would show preferential selectivity for cancer cells over normal or benign cells.

The significance of any effect shown against the PC3 cell line is that this cell line represents the type of cancer that is difficult to treat; androgen-independent metastatic prostate cancer. Importantly, when considering these novel compounds the question of mechanism is one that needs to be addressed. Members of the ALDH1A subfamily are known to play a regulatory role in the initiation and progression of tumors via their capacity to convert retinal to retinoic acid (RA). To further investigate a potential feedback loop, we treated 4 PCa primary prostate epithelial cell cultures (1xBPH, 1xBPH-PIN and 2x PCa) with atRA (100 nM) and found an increase in gene expression for ALDH1A3 while there was no apparent effect on the 1A1 isoform (Figure S5). Further studies using a larger sample size of primary PCa primary cells could provide a clearer understanding of how RA regulates ALDH isoform expression with implications for drug sensitivity.

**Figure 4:**
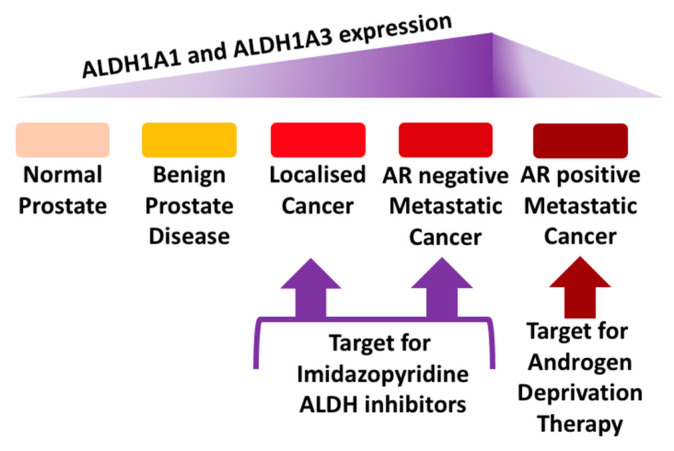
Proposed target for ALDH-targeting inhibitors including the imidazopyridine-based class of agent.

Inter-patient heterogeneity and distinct patterns of abnormal enzyme expression and regulation contribute to PCa patient relapse. Currently, hormone therapy remains the first choice for patients with advanced PCa, either alone or in combination with chemotherapy. The introduction of drugs to inhibit the biosynthetic steroidogenic pathway and androgen receptor have proved successful in treating PCa patients by extending overall survival rates, however, the majority of patients still relapse with CRPC. Resistance can develop in a number of ways and include androgen production outside the prostate microenvironment and harbouring sub-populations with tumor-initiating capacity within it. As we discussed previously [13,15,18,35], it is becoming apparent that new chemotypes and/or new drug combination strategies are required to target the heterogeneous PCa microenvironment more effectively [36]. Several studies have demonstrated that subpopulations of PCa that express high ALDH activity and possess stem-like properties are often aggressive, tumorigenic and metastatic [28]. PCSCs constitute a rare population of cells, which are quiescent and do not seem to express AR [37] and hence are less sensitive to M-phase cell cycle targeting taxanes such as docetaxel and cabazitaxel or drugs such as enzalutamide and abiraterone targeting the biosynthetic steroidogenic pathway. Accordingly, new therapies are required to target and eradicate the PCSC subpopulation and ALDHs have been proposed as a potential target [22,38]. Several medicinal chemistry efforts are underway which have proven the possibility of targeting specific ALDH isoforms. In this study, we profiled selected ALDHs and explored the potential for therapeutic intervention with our own recently discovered compounds in a panel of suitable prostate cell lines. Importantly, several compounds were shown to elicit potent antiproliferative activity and inhibition of colony-forming ability, with some correlation to the levels of ALDH1A1 and 1A3. Among the tested compounds, 3b exhibited nanomolar efficacy against the P4E6 and the PC3 cancer cell lines and 3d showed selective inhibition of PC3 colony formation. Therefore, these novel compounds have potential in terms of paving the way for treating aggressive forms of prostate cancer by means of a still untapped molecular target. LNCaP cells derived from prostate cancer lymph node metastasis are AR-positive and have low levels of the ALDH isoforms; these cells represent the prostate cancer cells that would respond to anti-androgen treatment. Crucially, in this study, the effect of the novel compounds on PC3 cells is significant because these represent the prostate cancer that currently has no successful treatment; androgen receptor-negative metastatic prostate cancer. Therefore, this study presents novel compounds that have the potential to target ALDH isoforms in the type of prostate cancer that requires novel treatments (Figure 4).

## Figures and Tables

**Table 1:** Showing antibodies used for Western Blots including source and concentrations used.

Company and Code	Antibody	Concentration
Cell Signaling Tech, D9J7R	ALDH1A1 1° Ab	1:500
Gene Tex, GTX110784	ALDH1A3 1° Ab	1:750
Abcam, AB9485	GAPDH 1° Ab	1:10,000
Cell Signaling Tech, 7074S	HRP-linked 2° Antibody	1:10,000

**Table 2:** Showing antibodies used for immunofluorescence including source and concentrations used.

Company and Code	Antibody	Concentration
Cell Signaling Tech, D9J7R	ALDH1A1 1° Ab	1:400
Gene Tex, GTX110784	ALDH1A3 1° Ab	1:400
Abcam, AB175471	Goat Anti-Rabbit IgG H&L (Alexa Fluor^®^ 568) 2° Antibody	1:10,000

**Table 3:** Anti-proliferative Activity of 6-Substituted-imidazo[1,2-*a*]pyridine Derivatives **3a–d**.

	EC_50_ (nM ^a^)					
N	R	P4E6	PC3	LNCaP	PNT2-C2	BPH1
**3a**	H	60.72	239.4	314.8	422.6	113.0
**3b**	4-F	4.038	70.92	240.0	217.0	41.47
**3c**	4-Cl	33.39	321.9	323.1	324.7	416.0
**3d**	3-CN	439.6	n.t. ^b^	7180.0	1718.0	351.0

^a^ EC_50_ values represent the concentration required to obtain half-maximal response. ^b^ Not tested.

## Supplementary Materials

The following are available online at https://www.mdpi.com/2227-9059/8/12/569/s1, Table S1: Taqman Assay Details. Figure S1: Synthetic procedure used to derive (substituted)imidazo[1,2-*a*]pyridine derivatives **3a–d**; Figure S2: qPCR analysis of ALDH expression in basal epithelial cells after subpopulation selection; Figure S3: Western Blot analysis of ALDH1A1 and ALDH1A3 expression in prostate cell lines and primary prostate epithelial cells. Figure S4: ImageJ analysis of fluorescence intensity profile of ALDH1A1 and ALDH1A3 staining. Figure S5: ALDH gene expression analysis of primary prostatic epithelial cells after atRA treatment.
