# Supplementary material for: Aldehyde Dehydrogenases and Prostate Cancer: Shedding Light on Isoform Distribution to Reveal Druggable Target
Source: Biomedicines. 2020 Dec 4;8(12):569. doi: 10.3390/biomedicines8120569 (PMC7761903; doi:10.3390/biomedicines8120569)
Supplement: Supplementary file 1 [file biomedicines-08-00569-s001.pdf]

Supplemental Information

Aldehyde dehydrogenases and prostate cancer: shedding light on isoform distribution to reveal druggable target.

Luca Quattrini, Maria Sadiq, Giovanni Petrarolo, Norman J. Maitland, Fiona M. Frame, Klaus Pors, Concettina la Motta.

Table of Contents

Table S1. Taqman Assay details.

Figure S1. Synthetic procedure used to derive (substituted)imidazo[1,2-*a*]pyridine derivatives **3a-d**.

Figure S2. qPCR analysis of ALDH expression in basal epithelial cells after subpopulation selection.

Figure S3. Western Blot analysis of ALDH1A1 and ALDH1A3 expression in prostate cell lines and primary prostate epithelial cells.

Figure S4: ImageJ analysis of fluorescence intensity profile of ALDH1A1 and ALDH1A3 staining.

Figure S5. ALDH gene expression analysis of primary prostatic epithelial cells after atRA treatment.

Table S1: Taqman Assay details

Target gene	TaqMan gene expression assay	Amplicon context Sequence containing probe sequence
**ALDH1A1**	Hs00946916_m1	5'-gcc gac ttg gac aat gct gtt gaa t-3'
**ALDH1A2**	Hs00180254_m1	5'-gat cat ccc atg gaa ctt ccc cct g-3'
**ALDH1A3**	Hs00167476_m1	5'-agg aga taa gcc cga cgt gga caa g-3'
**ALDH1B1**	Hs00377718_m1	5'-cct gct gca gag tgt cag cat gct g-3'
**ALDH2**	Hs01007998_m1	5'-agc agc ccg agg tct tct gca acc a-3'
**ALDH3A1**	Hs00964880_m1	5'-aag tca ctg aaa gag ttc tac ggg g-3'
**ALDH7A1**	Hs00609622_m1	5'-aaa atc tgg gca gat att cct gct c-3'
**RPLP0**	Hs04189669_g1	5'-gtc ctc gtg gaa ggc ccg gga ccg c-3'
**CA9**	Hs00154208_m1	5'-atc gct gag gaa ggc tca gag act c-3'

Figure S1. Synthetic procedure used to derive (substituted)imidazo[1,2-*a*]pyridine derivatives **3a-d**

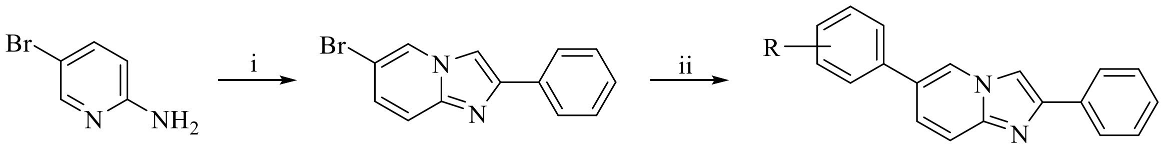

1

2

3a-d

3a: R=4-H
3b: R=4-F
3b: R=4-Cl
3c: R=3-CN

Reagents and Conditions:

i) 2-Bromo-1-phenylethan-1-one, K_2CO_3, MW; ii) (Substituted)phenylboronic acid, $Pd(OAc)_2$, PPh_3, K_2CO_3.

Figure S2. qPCR analysis of ALDH expression in basal epithelial cells after subpopulation selection.

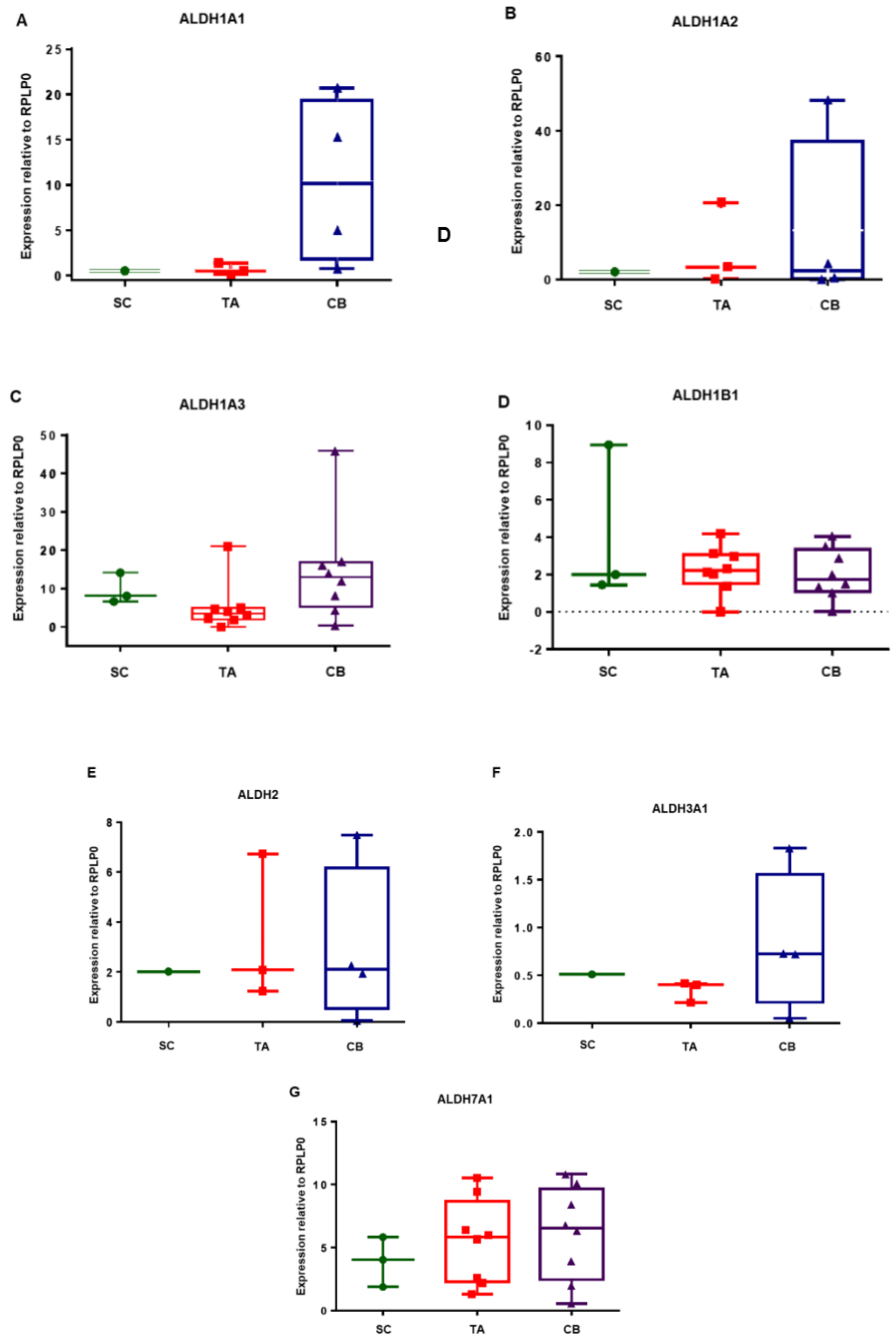

Gene expression of (A) ALDH1A1, (B) ALDH1A2 and (C) ALDH1A3, (D) ALDH1B1, (E) ALDH2, (F) ALDH3A1 and (G) ALDH7A1 using 2-dCT. RPLP0 was used as the control gene. SC- stem cell, TA- transit amplifying cell, and CB- committed basal cells. For ALDH1A1, 1A2, -2, and -3A1 statistical significance was assessed using the Mann Whitney U test for comparing unpaired groups for TA compared with CB, herein SC (n=1), TA (n=3) and CB (n=4). Due to only one sample for SC,

statistical test for this population was unobtainable. For ALDH1A3, -1B1 and -7A1 statistical significance was assessed using the one-way ANOVA for multiple comparisons of unpaired groups, TA (n=8) and CB (n=8) compared to SC (n=3), and no statistical significance was found. *Note difference in scale.

Figure S3. Western Blot analysis of ALDH1A1 and ALDH1A3 expression in prostate cell lines and primary prostate epithelial cells.

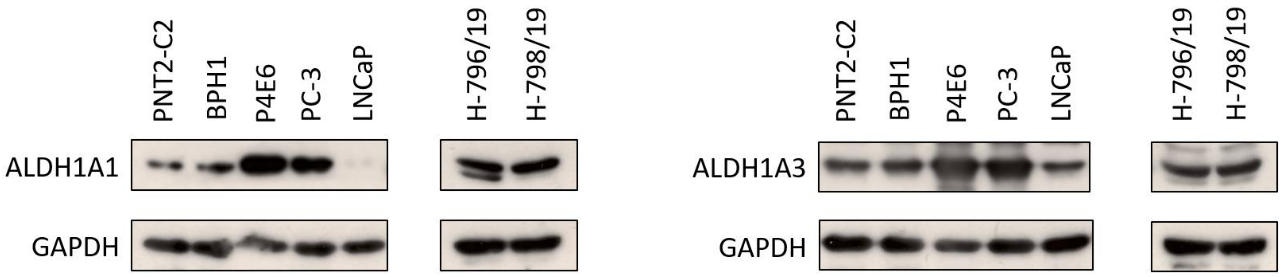

Analyses were carried out using ALDH1A1 and 1A3 specific antibodies in a panel of immortalised PCa cell lines (P4E6, PC-3, LNCaP), primary malignant lines (H796/19 and H-798/19) obtained from radical prostatectomies, a normal prostate epithelial cell line (PNT2-C2) and a benign hypertrophy cell line (BPH) obtained from transurethral resection of the prostate.

Figure S4: ImageJ analysis of fluorescence intensity profile of ALDH1A1 and ALDH1A3 staining.

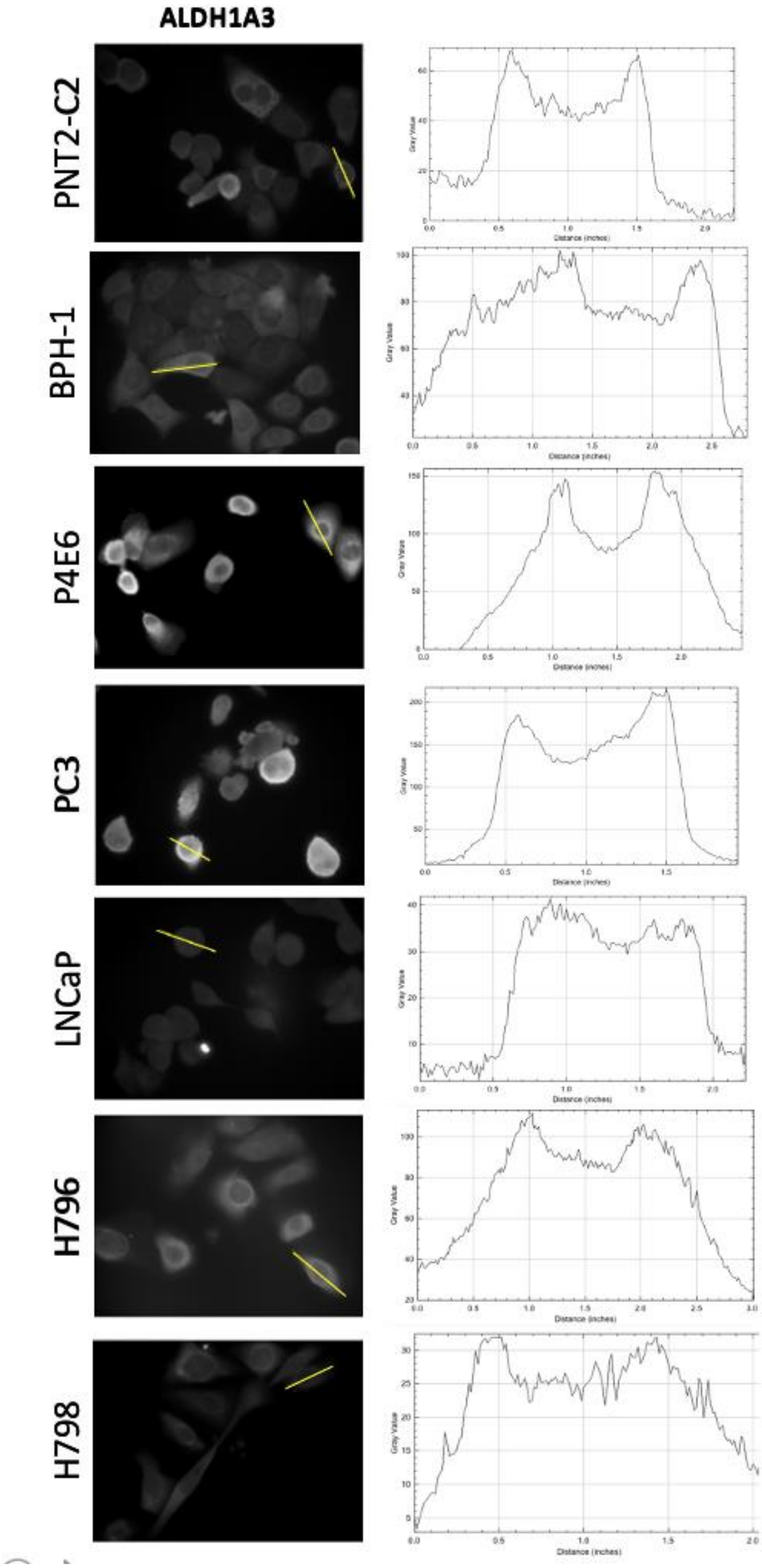

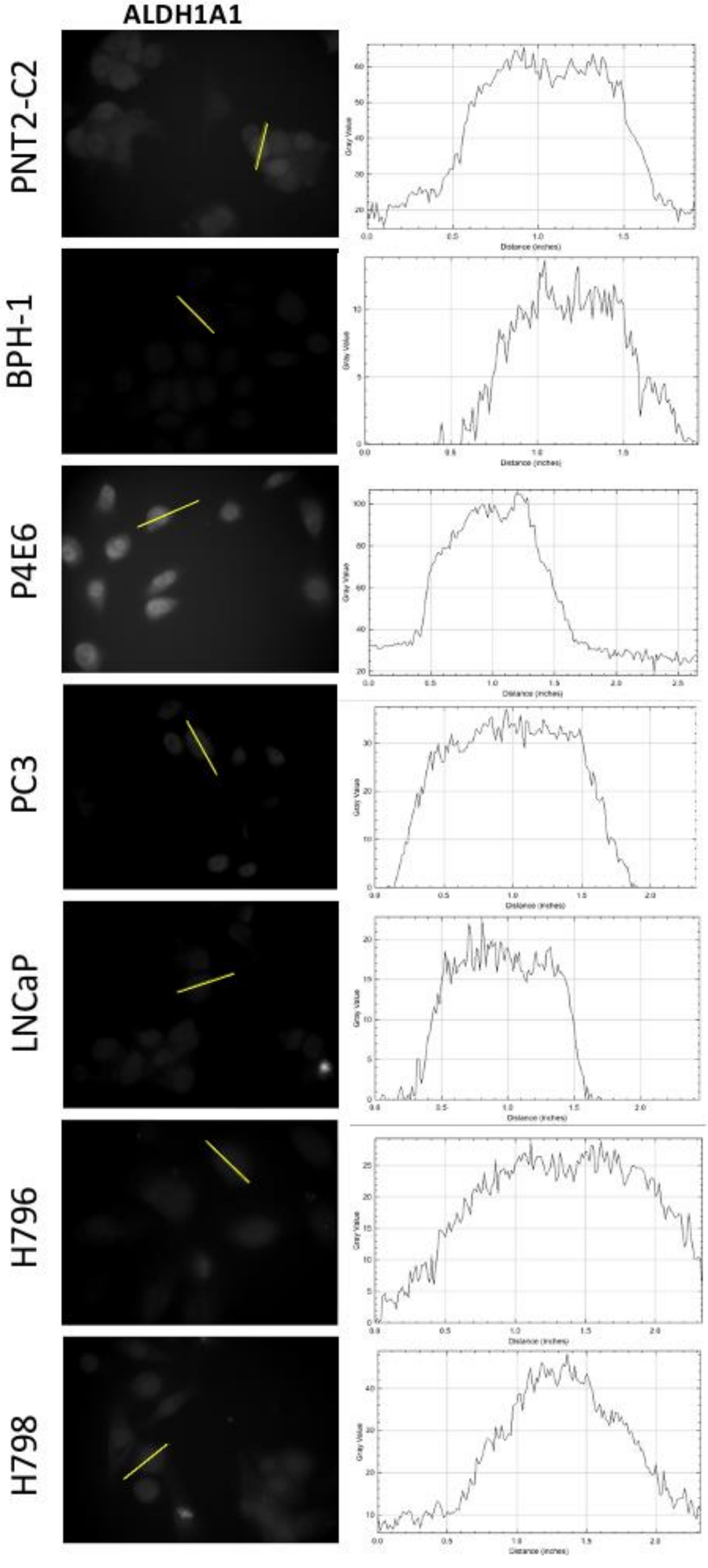
ALDH1A1
PNT2-C2
BPH-1
P4E6
PC3
LNCaP
H796
H798
Gray Value
Distance (inches)

	**ALDH1A3**		**ALDH1A1**	
**LOCATION**	**NUCLEUS**	**CYTOPLASM**	**NUCLEUS**	**CYTOPLASM**
PNT2-C2	+	+++	+	+
BPH-1	++	++++	+	+
P4E6	++	+++++	++++	++
PC3	++	+++++	+	+
LNCAP	+	+	+	+
H796	+	++++	+	+
H798	+	++	++	+

Images represent only the red channel (ALDH1A1 or ALDH1A3 staining). Images were imported into ImageJ and the yellow lines represent the area of analysis. The graphs represent fluorescent intensity on the y-axis and distance on the x-axis. Table shows summary of expression.

Figure S5. ALDH gene expression analysis of primary prostatic epithelial cells after atRA treatment.

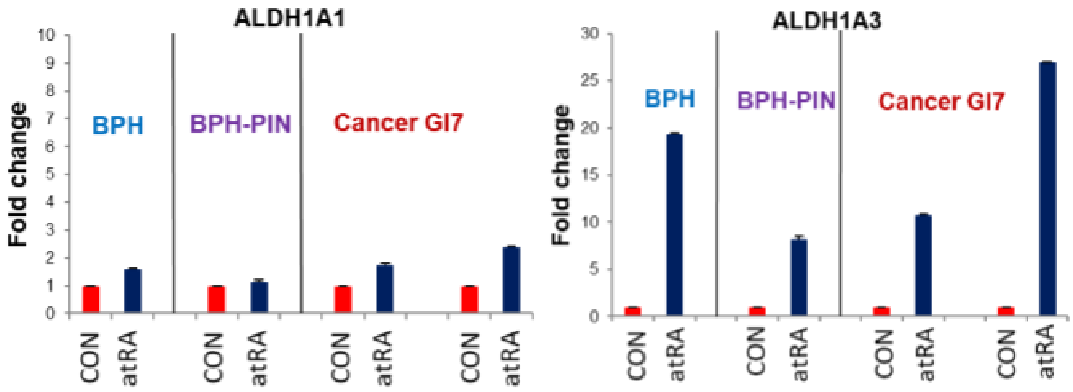

Fold change of ALDH1A1 and ALDH1A3 expression relative to untreated control. RPLP0 was used as the housekeeping gene. Analysis was performed by qPCR using 2-ddCT. Retinoic acid treatment was for 72 hours at 100nM. Samples studied were BPH, BPH-PIN (BPH sample but with signs of PIN) and cancer primary cells. Due to the requirement of a minimal of 3 repeats (n=3) to compare means of the groups, it was not possible to do apply a statistical test. Herein, BPH (n=1), BPH-PIN (n=1) and cancer (n=2) samples.
